# Path Planner for Autonomous Exploration of Underground Mines by Aerial Vehicles

**DOI:** 10.3390/s20154259

**Published:** 2020-07-30

**Authors:** Carlos Rubio-Sierra, Diego Domínguez, Jesús Gonzalo, Alberto Escapa

**Affiliations:** Aerospace Engineering Area, Universidad de León, Campus de Vegazana s/n, 24071 León, Spain; ddomf@unileon.es (D.D.); jesus.gonzalo@unileon.es (J.G.); alberto.escapa@unileon.es (A.E.)

**Keywords:** path planner, autonomous exploration, underground mines, aerial robot, LIDAR-based navigator, obstacle avoidance

## Abstract

This paper presents a path planner solution that makes it possible to autonomously explore underground mines with aerial robots (typically multicopters). In these environments the operations may be limited by many factors like the lack of external navigation signals, the narrow passages and the absence of radio communications. The designed path planner is defined as a simple and highly computationally efficient algorithm that, only relying on a laser imaging detection and ranging (LIDAR) sensor with Simultaneous localization and mapping (SLAM) capability, permits the exploration of a set of single-level mining tunnels. It performs dynamic planning based on exploration vectors, a novel variant of the open sector method with reinforced filtering. The algorithm incorporates global awareness and obstacle avoidance modules. The first one prevents the possibility of getting trapped in a loop, whereas the second one facilitates the navigation along narrow tunnels. The performance of the proposed solution has been tested in different study cases with a Hardware-in-the-loop (HIL) simulator developed for this purpose. In all situations the path planner logic performed as expected and the used routing was optimal. Furthermore, the path efficiency, measured in terms of traveled distance and used time, was high when compared with an ideal reference case. The result is a very fast, real-time, and static memory capable algorithm, which implemented on the proposed architecture presents a feasible solution for the autonomous exploration of underground mines.

## 1. Introduction

Aerial robots are an exceptional solution for outdoor exploring and mapping, but in an underground environment they present significant challenges that have delayed them from being widely used. The research interest in this field is very high due to its large potential for saving costs, and even human lives, when it addresses the exploration of dangerous areas. This interest is well illustrated by the Defense Advanced Research Projects Agency (DARPA) Subterranean Challenge [1]. It is rewarded with $2 million dollar prizes and reproduces common challenges for automated underground exploration (a global navigation satellite system (GNSS) denial environment, the unfeasibility of radio communications, the navigation in narrow passages and sometimes in dusty or wet conditions). Although knowledge of subterranean spaces has tremendous value across a number of applications [2], mines are of particular interest for automatic exploration (large number and horizontal size and inherent risks: poisonous gases, roof collapses, water, etc.).

When it comes to developing the exploration robot, no single design could be applicable to every conceivable subterranean space, which is why a myriad of different ground vehicles have been proposed in the past [2,3]. Aerial robots, although harder to control and more prone to suffer accidents due to the unstable nature of vertical flight, provide unbeatable advantages like movement speed, independence of ground surface characteristics (sand, water, etc.) or obstacle avoidance capability. Furthermore, passing through narrow passages is a very common requirement to explore underground sites, thus small robots are required. Additionally, the exploration space is quite large, thus a careful design analysis should be conducted to allow adequate flight time and payload capacity while keeping the battery small enough. Consequently, this limits the weight and consumption of the on-board sensors and computing devices.

In recent years, different proposals have been made that use aerial robots, usually employing various kinds of high-end on-board sensors (e.g., thermal cameras, 3D lidars, and radars) to reach reliable navigation capabilities in the dark and featureless underground environments that challenge the state estimation process [4,5]. Although cameras have been widely used in robotic exploration, since they provide rich data about the surroundings which is invaluable for robust navigation, they present relevant challenges in non-illuminated or visually homogeneous environments. Thus, previous works with aerial robots commonly use some kind of range finding technology as the primary sensor, whether it be LIDAR 2D [6], LIDAR 3D [7] or sound navigation and ranging (SONAR) [8]. They are always combined with an inertial system unit (INS) and, on some occasions, assisted by visual stereo cameras [4] or optical flow sensors (required illumination provided by on-board lights) [9]. Besides, another difficulty is that there are no reliable maps available for most underground missions, so there is a need to discover the geometry of the mine while exploring it for the first time. This entails that the sensors are used according to the classical simultaneous localization and mapping (SLAM) approach [10].

As there is no pre-existing map, it is not possible to predefine a motion plan. The robot should avoid the obstacles and make motion decisions based only on its on-board sensors. Local decisions are needed but, in the presence of circular galleries (abusing terminology, we will not make distinctions among the meaning of gallery, tunnel, passage, etc., all referring to the geometrical connections of a single level underground mine. A precise definition of them in the context of mine research can be found at [11]. Besides, we will not differentiate between labyrinth and maze, meaning the path structure of the underground mine), the robot would be trapped in the absence of some kind of global knowledge [2]. To achieve this, global awareness is then needed to guarantee that the robot explores the mine galleries and returns to base.

The process to define robot movements along the mine (path planning) varies from global to local strategies. When global planning is used, it is augmented with some kind of local adjusting, e.g., dynamic insertion of new obstacles [2], a wavefront-based global planner with local vector field histogram analysis in [12] or a predefined path with local checkpoint marks [8]. The alternative is making use of the pure local planning, which usually requires less processing resources.

Different local path planning strategies can be defined: Deterministic tree-based, assigning different branch gains to select the next direction [4], random tree [13], or probabilistic road map [14]. Besides, the path planning process should be complemented with an obstacle avoidance mechanism. Different methods have been tested, such as artificial potential fields (APF) [15,16] where obstacles generate a repulsive force, and sector-based [17,18] or gap-based methods [19] that look for the best open space to decide the next movement.

The purpose of this work is to define a path planner algorithm that permits the autonomous exploration of a large set of single level mining galleries by an unmanned aerial vehicle. The galleries can present a small slope and its section is assumed to be near vertical. The stated conditions of unfeasibility of GNSS and radio communications are assumed. To successfully conduct the mission, a LIDAR sensor with integrated SLAM is proposed as a simple solution, so the article focuses on solving the path planning and obstacle avoidance problems.

Global path planning is not an option because the mine map is unknown a priori. Previously mentioned local strategies store a dynamic logical map, but it can be difficult to build if its elements (e.g., intersections) need to be derived from the noisy and unstable LIDAR scans. Also, the map size increases with the flight time, precluding or at least complicates an entire implementation using only static memory, which is always preferred in real-time applications. Finally, the logical map can be broken if there is a significant error in the estimate of the location. This can occur when traversing long and constant section galleries where there are not good references for the SLAM algorithm.

To prevent these problems, a dynamic planning is proposed based on exploration vectors, a novel variant of the open sector method with reinforced filtering [17]. In particular, it enhances previous approaches by introducing a two-stage filtering combined with hysteresis. First, the sectors are grouped in representative exploring vectors, then a low pass filtering of the azimuth and distance is performed, and finally, a hysteresis filter is applied in an analogous way as in radar processing. At the same time, the required global awareness is maintained using a visited zone grid. Obstacle avoidance is performed primarily by the directional guide of the exploration vectors and secondary by a reactive yaw correction near the walls. Finally, labyrinth solving (intricate mine galleries are genuine labyrinths) is done using a modified Tremaux’s [20] algorithm that does not need to store the global logical map.

The proposed algorithm integrates the concept of exploration vectors, the two-stage filtering, the navigator logic, and the angle-based obstacle avoidance into a whole system, providing an appropriate solution for the exploration problem. To our knowledge, this approach is not hitherto present in the literature. Furthermore, recent researches related to mine exploration using aerial vehicles [4,5,9] just rely on local approaches and do not consider bifurcations and labyrinth solving, in contrast to our solution.

The path planner algorithm is validated thanks to a hardware-in-the-loop (HIL) simulator. A synthetic LIDAR model is created to generate realistic range data. A complete 6 Degrees of Freedom (DoF) of the air vehicle is also needed, combining the well-known Ardupilot controller with simple navigation laws fitted to the quasi-horizontal structure of the galleries. In this way, some state variables are controlled by an autopilot, like the flying height, and the path planner just relays to a 3 DoF system. It is described by the position of the center of the mass in x and y, and the yaw of the vehicle, forming its pose.

From a formal point of view, the proposed algorithm can also be applied when the exploration is performed by ground vehicles. However, its practical implementation is favored for aerial vehicles, since their navigation is independent from ground surface features. In this way, height control allows to keep a mid-level horizontal position along the tunnels, making the processing of LIDAR data easier.

This paper is organized as follows: Section 2 describes the path planner algorithm, which comprises the laser scan processing in Section 2.1, the visited zone tracking in Section 2.2, the navigation module logic in Section 2.3, and the obstacle avoidance in Section 2.4. Section 3 explains the experimental setup used to validate the design, introduces the hadware in the loop simulator in Section 3.1, its configuration in Section 3.2, and performs a sensitivy analysis of the algorithm execution times in Section 3.3. Section 4 shows the simulation results, introducing the study cases in Section 4.1, and presenting the results for canonical situations in Section 4.2 and complete galleries in Section 4.3. Finally, in Section 5 and Section 6 the discussion and conclusion of this work are presented.

## 2. Path Planner Algorithm

As we have pointed out in the Introduction, our aim is to design a path planner algorithm that allows the autonomous exploration of mine galleries. This exploration is performed by an aerial robot equipped with a LIDAR sensor with SLAM capabilities.

**Figure 1:**
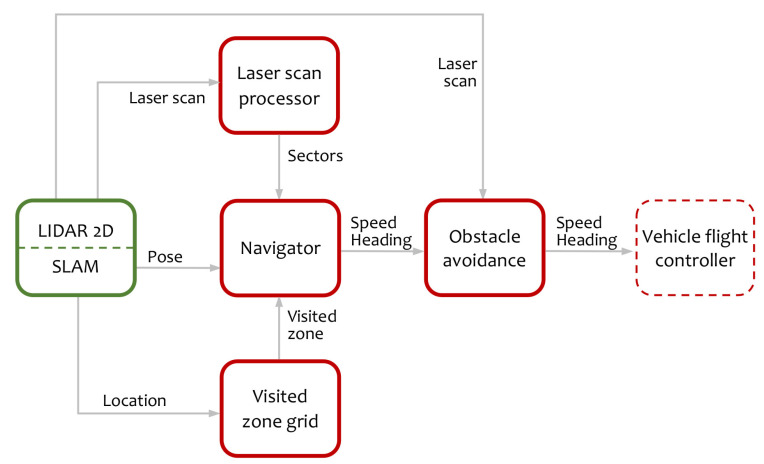
Top-level information flow diagram. In red are represented the algorithm components, in green the sensor, and in dashed line the vehicle flight controller.

The algorithm comprises four principal components represented in Figure 1. The first one is the laser scan processing that provides a sector representation of the environment based on the LIDAR measurements. The second component is a grid structure to track visited zones. Next is the navigation module that is responsible for the movement decisions and, finally, the obstacle avoidance module. The sensor provides the raw laser scans relative to a LIDAR axes set and the pose of the vehicle with respect to a SLAM reference system. Laser scans are sets of laser points, each one characterized by its azimuth and distance. Laser scans are used by the laser scan processor to obtain a sector representation of the obstacles around the vehicle. Using this representation and the knowledge of the visited zone, the navigation module establishes the yaw and speed of the robot. Finally, an obstacle avoidance component modifies the direction of the movement to avoid collisions and to help vehicle stay centered in the tunnel.

### 2.1. Laser Scan Processing

The first task of the algorithm is to obtain a simplified representation of the obstacles around the vehicle. Dynamic planning is performed based on this representation in real-time using a *Gap Method*, *AGAP*, strategy [21].

For the target of exploring a labyrinth of tunnels with a near vertical section, it is good enough to use a 2D scan around the vehicle. This scan can be obtained using a rotary LIDAR sensor installed at the top of the vehicle while it is flying at an intermediate height inside the tunnel.

The sample rate and the rotation speed of the LIDAR scanner should be considered in relation to the vehicle dynamics to decide to ignore individual sample delays. Available mappers impose a low vehicle translational speed and provide a high sample rate. Based on this, the LIDAR scanner is considered fast enough to process a complete laser scan without compensating individual sample delays. Furthermore, asynchronous readings of complete laser scans were used in the experimental setup and the observed performance of the overall system was satisfactory.

The LIDAR is fixed in the vehicle, centered in its horizontal plane, and aligned with the forward movement direction. So the LIDAR reference system is centered in the sensor ($O_{b}$), with x axis ($x_{b}$) pointing to the front of the vehicle and y axis ($y_{b}$) pointing to the right. The vehicle maintains a quasi-horizontal attitude with slight pitch and roll angles, so a 2D reference system is enough for the algorithm development.

A complete laser scan contains a list of points. Each of them is characterized by an azimuth $\phi_{P}$ relative to $O_{b}X_{b}$, and a range, distance to $O_{b}$, $\rho_{P}$. Two extreme conditions occur with obstacles too far or too close to the sensor. When the obstacle is too far, no laser point is generated in that direction, so the number of points received in one scan is not constant, and can even be zero. When an obstacle is too close to the sensor, the points are marked as invalid and discarded as the LIDARs cannot measure very small distances.

**Figure 2:**
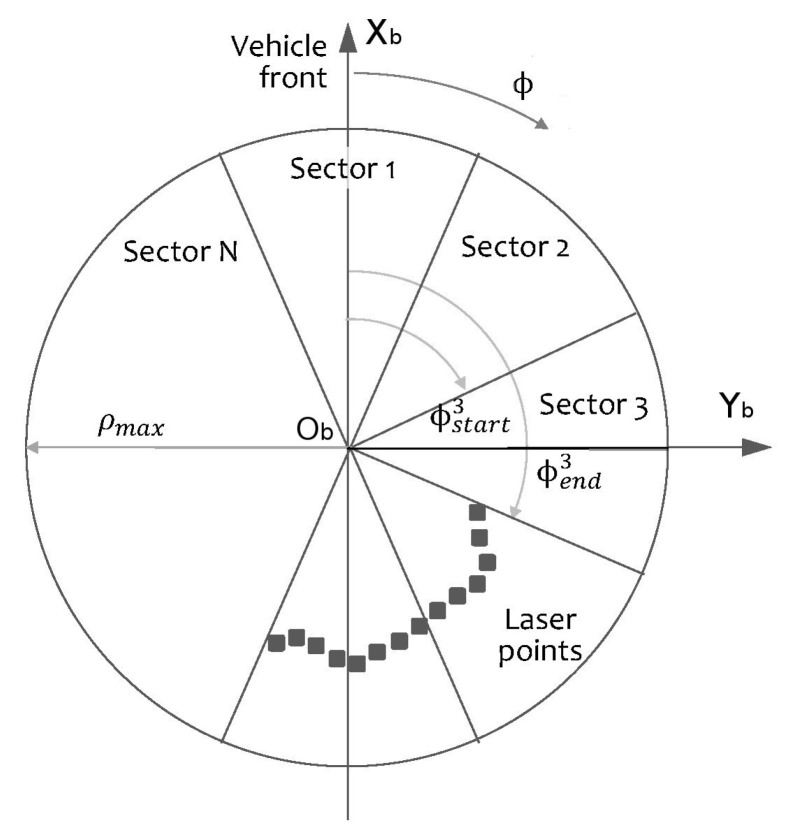
Constant angle sectors for obstacle identification.

The selected simplified representation of the surrounding obstacles is sector-based. Instead of using variable angle sectors like in [17], a fixed angle sector representation was selected. Fixed data structures are favored in this work as the resulting implementation is simpler and allows to implement the algorithm using only static memory. The entire 360° azimuth range is divided into *N* identical sectors, as shown in Figure 2. If the sector with *n* = 1 is bisected by the axis $x_{b}$, we can define the limiting angles

(1) $$\phi_{start}^{n}=\left(2n-1\right)\frac{\pi }{N},\;\phi_{end}^{n}=\left(2n+1\right)\frac{\pi }{N},\;n=1,2,\ldots ,N$$

where *n* is the sector index.

The first step is to group the laser scan points in a set of sectors using the sector angle limits. A point *P* belongs to sector $S^{n}$ if

(2) $$P\in S^{n}\;\mathrm{if}\;\phi_{P}>\phi_{start}^{n}\;\mathrm{and}\;\phi_{P}\leq \phi_{end}^{n}.$$

For each sector the algorithm should calculate a representative distance to the nearest obstacles. As LIDAR sensor measurements can be affected by dust, reflections, and noise, some kind of probabilistic threshold is needed. A threshold based on the polar histogram was selected and performed well in the simulation tests.

For a sector with no points or with a very small number of points, the maximum scan distance $\rho_{max}$ is set, meaning that there are no obstacles in this sector. The reason for this rule is to avoid dust problems inside the tunnel, otherwise sporadic false detections caused by the dust will be considered as real obstacles.

If there are enough points in the sector only a fraction of them, the nearest ones, are used for the distance calculation. Simulation tests using just one third of the samples provided good results. Let us call $\Lambda^{n}$ as the set $J^{n}$ nearest points of the sector *n*: $P_{1}$,$P_{2}$, ..., $P_{n}$. The mean distance of $\Lambda^{n}$ is taken as the representative range of the sector. If we denote |S| as the range of a sector, ∠S as the azimuth of the angle bisector, *J* the number of the nearest points in the sector, and $J_{min}$ the minimum number of points to be considered, we have

(3) $$|S^{n}|=\left\{\begin{array}{cc} \rho_{max} & \mathrm{if}\;\;J^{n}<J_{min}\\ \frac{1}{J^{n}}\left(\rho_{1}+\rho_{2}+\cdots +\rho_{J^{n}}\right) & \mathrm{if}\;\;J^{n}\geq J_{min}, \end{array}\right.$$

(4) $$∠S^{n}=\frac{1}{2}\left(\phi_{start}^{n}+\phi_{end}^{n}\right),$$

This simplified representation of the surrounding obstacles based in constant angle sectors can be used directly for obstacle avoidance in the flight control unit. Moreover, the path planner uses the sectors to evaluate the accessible exploration directions.

Next we will introduce the exploration vector Ω. It is defined as a suitable direction to explore from the present vehicle position. The search for exploration vectors starts selecting the sector with the largest range from the last laser scan. If the range of this sector is long enough, this sector will be the seed for a new exploration vector.

Next, the sectors at the left and the right of the seed sector will be evaluated to check if they belong to the same mine passage. One criterion that performed well in the simulated tests was to use a fraction of the range of the seed sector, but other criteria based on fixed limits can be used. All the sectors that meet the criterion, plus the seed sector, form a group from where the exploration vector will be calculated.

The exploration vector azimuth is calculated as the weighted combination of the azimuth of the sectors of the group. The resulting azimuth is towards the more open part of the passage. The modulus of an exploration vector is set to be the sum of the ranges of the sectors of the group. This makes it possible to provide an estimation about how big the mine passage is

(5) $$|\Omega_{k}|=\sum_{m=1}^{M}\left|S_{m}\right|,$$

(6) $$∠\Omega_{k}=\frac{\sum_{m=1}^{M}∠S_{m}\left|S_{m}\right|}{|\Omega_{k}|},$$

where *M* is the number of sectors within the same passage.

The seed sector, the continuation sectors and the two boundary sectors at the left and at the right are marked as processed at this point, the algorithm continues evaluating the remaining sectors until no sector can be used as the seed sector.

Once exploration vectors are calculated, special care should be taken to maintain these vectors as stable as possible. They are the root of the movement commands for the vehicle. Instability in the exploration vectors can cause oscillations and erratic movements.

Two strategies are jointly used to provide this stability: low pass filtering and hysteresis. Hysteresis is widely used in radar processing to provide stable tracks [22]. In those applications a tentative radar track becomes active only when confirmed by several consecutive detections.

The azimuth and the range of the exploration vectors are filtered using configurable first-order low-pass filters

(7) $${\left|\Omega_{k}\right|}^{t}={\left|\Omega_{k}\right|}^{t-1}+\alpha_{1}\left(|\Omega_{k}{|}^{t}-{\left|\Omega_{k}\right|}^{t-1}\right),$$

(8) $${\left(∠\Omega_{k}\right)}^{t}={\left(∠\Omega_{k}\right)}^{t-1}+\alpha_{2}\left[{\left(∠\Omega_{k}\right)}^{t}-{\left(∠\Omega_{k}\right)}^{t-1}\right],$$

where $\alpha_{1}$ and $\alpha_{2}$ are the filter coefficients and *t* represents the discrete laser scan time.

**Figure 3:**
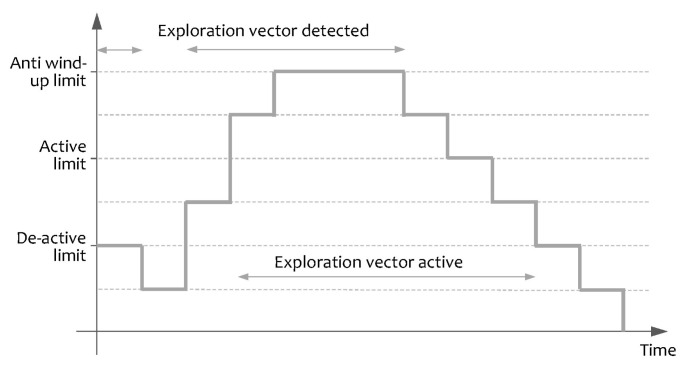
Hysteresis algorithm applied to the exploration vectors.

Hysteresis controls the activation and deactivation of the exploration vectors to prevent flicker. One exploration vector is considered active only if it is detected and confirmed by several laser scans. Once active, the vector maintains a refresh counter and needs several non-detected laser scans to be deactivated. The hysteresis algorithm is illustrated in Figure 3.

**Figure 4:**
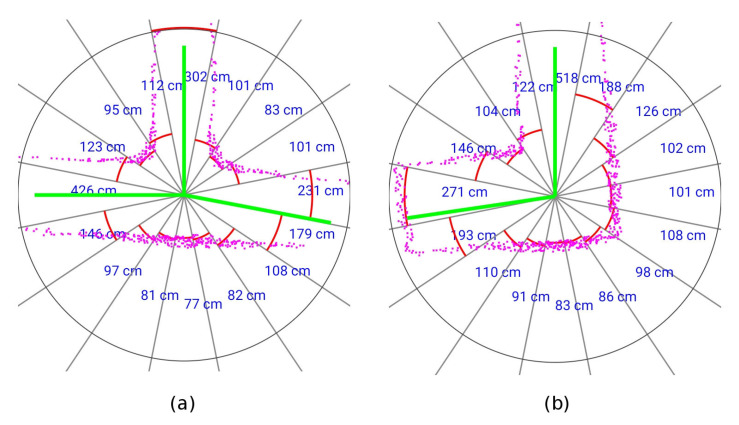
Scan processing result. (**a**) Three-way intersection. (**b**) Tunnel corner.

An example of the information at the end of the scan processor stage, obtained in the simulated tests, is represented in Figure 4. The magenta points are the individual laser scan points. The red arcs in the figure indicate the range of each sector, this range approximates the distance to the nearest obstacle. The green lines are the exploration vectors. These vectors show the mine passages ready to explore.

### 2.2. Visited Zone Tracking

Although the proposed system aims to enable a navigation strategy based on real-time LIDAR data, some kind of global awareness is required, and an efficient technique is needed to track the visited zones by the vehicle. Based on the knowledge of the visited area, the navigation module chooses which exploration vectors should be followed.

The sensor includes SLAM capability and provides estimation of the location and the attitude in the horizontal plane (*x*, *y*, and yaw angle). The SLAM reference system is set at device startup or after a map reset command, so the origin and the axis orientation is considered arbitrarily and all the calculations should be done relative to this original setup. Let us call Os the origin of the sensor coordinate system, and $x_{s}$ and $y_{s}$ its cartesian axes. The yaw angle is defined as positive in the colckwise direction, relative to $O_{s}X_{s}$. The information needed to track the explored area is the location of the vehicle in this coordinate system.

The navigation module needs to store the visited locations to be able to explore the mine tunnels without getting trapped in a loop. Storing individual locations can degrade the system performance as time goes up, so a fixed size grid scheme is preferred. Cartesian grids along with relational topological representations were broadly used to store robot maps [23]. Triangular, hexagonal, or squared shapes can be used to tessellate the grid.

Regardless of the shape of the cells, one problem arises when the location is very close to the boundary of a cell. When this happens, a location slightly different from the visited one is considered as not visited. To avoid this boundary problem multiple cells can be marked as visited around the location of the vehicle or only one cell marked but several cells checked when testing if a position is visited. This second strategy is used.

The time when a location was visited is also useful for the identification of the mine tunnels pattern. Let us call the less explored position or positions from a set as the positions that were never visited or if all were visited then the position with older visited time.

Using a square grid of side *s*, a point P is associated to a cell $C_{P}=\left(C_{x},C_{y}\right)$ with

(9) $$C_{x}=\mathrm{floor}\left(\frac{x}{s}\right),\;C_{y}=\mathrm{floor}\left(\frac{y}{s}\right).$$

The cells checked to test if a location was visited are $C_{P}$ and its first neighbors, i.e., the cells that share at least one vertex with $C_{P}$.

In an environment with constant width tunnels, the cell side *s* can be selected to guarantee that all the width of the tunnel is marked as visited. This election averts traverse the same tunnel several times with different lateral offsets.

Explored area is evaluated only to check if an exploration vector is visited. Evaluation is done at a fixed distance of the vehicle independently of the modulus of the exploration vector. This distance is called the exploration radius δ. The exploration radius should be long enough to not be affected by the present location of the vehicle, otherwise it will be considered as recently visited and will never be explored.

Let us consider some random motion of the vehicle around its present location in the direction of the exploration vector, characterized by a random variable M with zero mean and variance $\sigma^{2}$. That variance can be estimated by low pass filtering motion records.

Assuming that the exploration vector is pointing forward along any of the square diagonals with σ<s, the most unfavorable situation arises when the current location is very close to the respective bottom vertex. It entails that the limit of the current position extends two cells diagonals along the direction of the exploration vector. Hence, the exploration radius would be equal to $2\sqrt{2}s$.

Besides, we should consider the random motion of the vehicles. So, that quantity must be augmented by *k* times the standard deviation σ of the random variable M

(10) $$\delta =2\sqrt{2}s+k\sigma .$$

For example, if M can be modelled by a normal Gaussian distribution $N(0,\sigma^{2})$, taking k=3 corresponds to a confidence about 99.7%. Equation (10) represents the most conservative value since the location of the vehicle in the cell is not always in the worst position.

**Figure 5:**
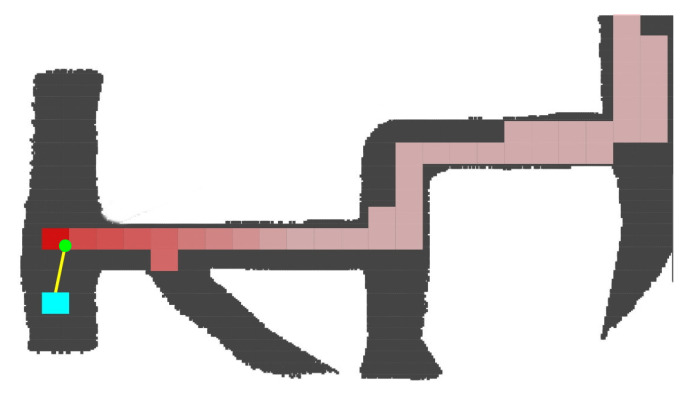
Visited zone tracking.

Figure 5 represents in gray the scanned mine tunnels and in red the visited zones. The gradual color changes represent the time when the vehicle occupied that position, the clearer ones being the first visited. The current vehicle position is at the small green circle, and its orientation in the sensor reference system is towards the yellow line. The cyan squared box is at the exploration radius distance from the vehicle and is where the algorithm is evaluating whether an exploring vector is visited or not.

### 2.3. Navigation Module

The navigation module provides the speed and direction of the next vehicle movement. It is based on the sectors and exploration vectors derived from the LIDAR measurements but also considers the visited zone to avoid re-exploring the same tunnels and getting trapped in circular paths.

Since the main goal of this research is to design a path planner, we have implemented simple but effective navigation laws that determines the target speed and the correction for yaw angle, which is the difference between the target and current yaw angles.

Speed target is discretized into three levels: high speed, low speed, and zero speed. High speed is used when advancing along a tunnel, low speed is used when the vehicle is approaching a wall and zero speed is used when the vehicle needs to rotate to explore a new direction. Each speed level value is realized by a proper selection of the vehicle pitch angle, since motor response is driven by the altitude autopilot.

The navigation module constitutes a state machine with four states: advance, block, rotate, and stop. In advance state it executes Algorithm 1 that is used when the vehicle progresses along a tunnel. During block state runs Algorithm 2, used when the vehicle gets close to a wall and needs to approximate it to scan new directions. Rotate state with Algorithm 3 is used when it is needed a big change in the direction and finally, it executes Algorithm 4 in the stop state for the cases that there is not any active exploration vector.

In the advance state, the navigation algorithm follows the current tunnel. Progressing along a tunnel is determined by the continuation vector, which is the exploration vector more aligned with the current direction provided the change of direction is below a fixed tolerance. In this state the navigator follows the continuation vector which allows progressing along straight and curved tunnels. In addition, in this state the algorithm detects if other directions are less explored, in an analogous sense as less explored positions, and divert to follow them.

When a front wall is detected, the navigation module switches to the block state. The vehicle will move slowly until it gets close to the wall to detect other possible directions. If during the approach to the wall a continuation vector is detected, the navigation module reattaches to it and continues moving in that direction.

In the rotate state, the speed is set to zero, and yaw angle is set to orientate the vehicle towards the less explored direction. If there is not any exploration vector the vehicle stops, but there should always exist at least one exploration vector; the vector pointing back.

The stop state is similar to the rotate state; the vehicle remains there until a new exploration vector is detected. The vehicle will continue towards the less explored vector.

According to this description, the identification of the mine tunnels pattern is solved using a modified Tremaux’s algorithm. The initial search is done exactly the same as in the standard algorithm described by Tarry in 1895 [20]; deep nodes first. The rule to prevent revisiting the same corridor several times differs as now it is not a hard rule, it is mimicked with the *less explored* condition. This difference allows returning to the base location without the need for a logical global map. The counterpart of this simplification is that the mine gallery is not always fully explored, which it is a common issue for local methods [2].
**Algorithm 1:** Advance state
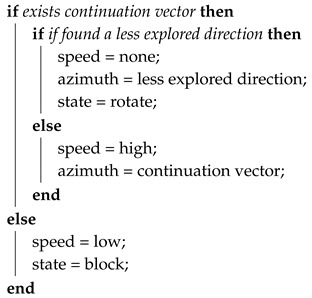


**Algorithm 2:** Block state

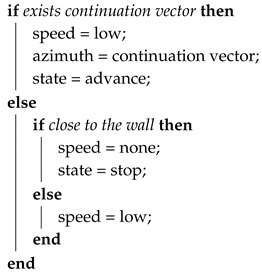



**Algorithm 3:** Rotate state

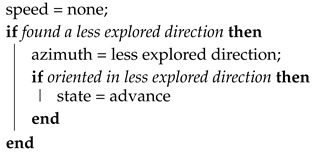



However, as the exploration vectors are calculated at each intersection, there is enough information to detect non-explored tunnels. These vectors can be stored in the vehicle memory, and after the flight, analyzed to determine the unexplored paths.
**Algorithm 4:** Stop state
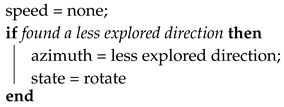


### 2.4. Obstacle Avoidance

An obstacle avoidance algorithm improves the performance of the system because in addition to avoiding collisions, it keeps the vehicle more centered inside the tunnel. The exploration vectors also tend to center the vehicle due to the weighted azimuth calculated in Equation (6), but this centering action can be insufficient in some situations.

The Artificial Potential Field (APF) approach as described in [15] is used, but with the difference that instead of creating a potential field with gradient-based forces, the field is created to provide directly angular corrections in the yaw angle.

**Figure 6:**
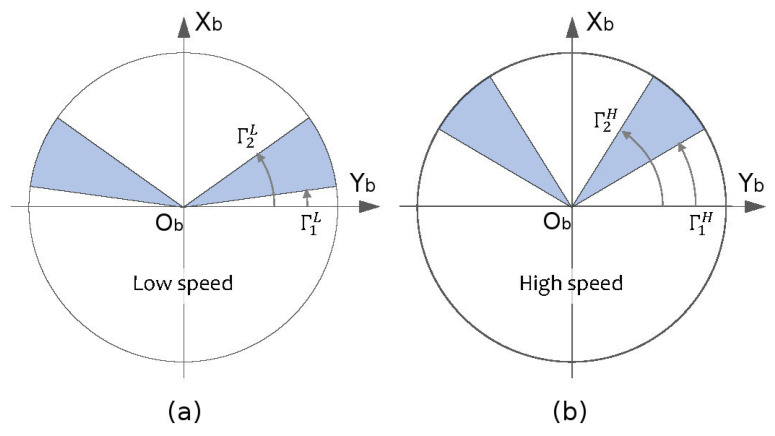
Obstacle avoidance sectors. (**a**) Definition for low speed mode. (**b**) Definition for high speed mode.

When the vehicle is in movement, two sectors are considered for obstacle avoidance: one sector left ahead $S^{l}$ and other right ahead $S^{r}$ of the vehicle. Start and end angles for these sectors are fixed depending on the vehicle speed. For low and high speed modes these angles are designated as $\Gamma_{1}^{H}$, $\Gamma_{2}^{H}$, $\Gamma_{1}^{L}$ and $\Gamma_{2}^{L}$. To make more direct their interpretation, they are measured from the axis $OY_{b}$ in an anticlockwise way as shown in Figure 6. For high speed the angles are larger than in the low speed case as we need to anticipate obstacles that are further away.

The representative distance of the sectors $S^{l}$ and $S^{r}$, denoted respectively as $\kappa^{l}$ and $\kappa^{r}$, is calculated just the same as described in the laser scan processing Section 2.1: First by filtering the points inside the sector angle span, then selecting the nearest ones, and finally calculating the mean value of them. Both sectors can provide a correction to the target yaw angle. The corrections are calculated independently and the avoidance correction angle $\Delta_{a}\psi$ is the sum of the left and right ones

(11) $$\Delta_{a}\psi =\Delta_{a}^{l}\psi +\Delta_{a}^{r}\psi .$$

Let us introduce the left reactive distance $\tau^{l}$ as the distance to the left wall where the obstacle avoidance becomes active, and analogously the right reactive distance $\tau^{r}$. The left avoidance correction angle $\Delta_{a}^{l}\psi$ and the right one $\Delta_{a}^{r}\psi$ are given by

(12) $$\Delta_{a}^{l}\psi =\left\{\begin{array}{ccc} 0 & \mathrm{if} & \kappa^{l}>\tau^{l}\\ \frac{\pi }{2}\;\cos\left(\frac{\pi }{2}\frac{\kappa^{l}}{\tau^{l}}\right) & \mathrm{if} & \kappa^{l}\leq \tau^{l}, \end{array}\right.\;\Delta_{a}^{r}\psi =\left\{\begin{array}{ccc} 0 & \mathrm{if} & \kappa^{r}>\tau^{r}\\ -\frac{\pi }{2}\;\cos\left(\frac{\pi }{2}\frac{\kappa^{r}}{\tau^{r}}\right) & \mathrm{if} & \kappa^{r}\leq \tau^{r}. \end{array}\right.$$

where $\kappa^{l,r}$ are the representative distance of the left or right sector. If $\tau^{l}$ and $\tau^{r}$ are equal, we can define only one value τ named reactive distance.

**Figure 7:**
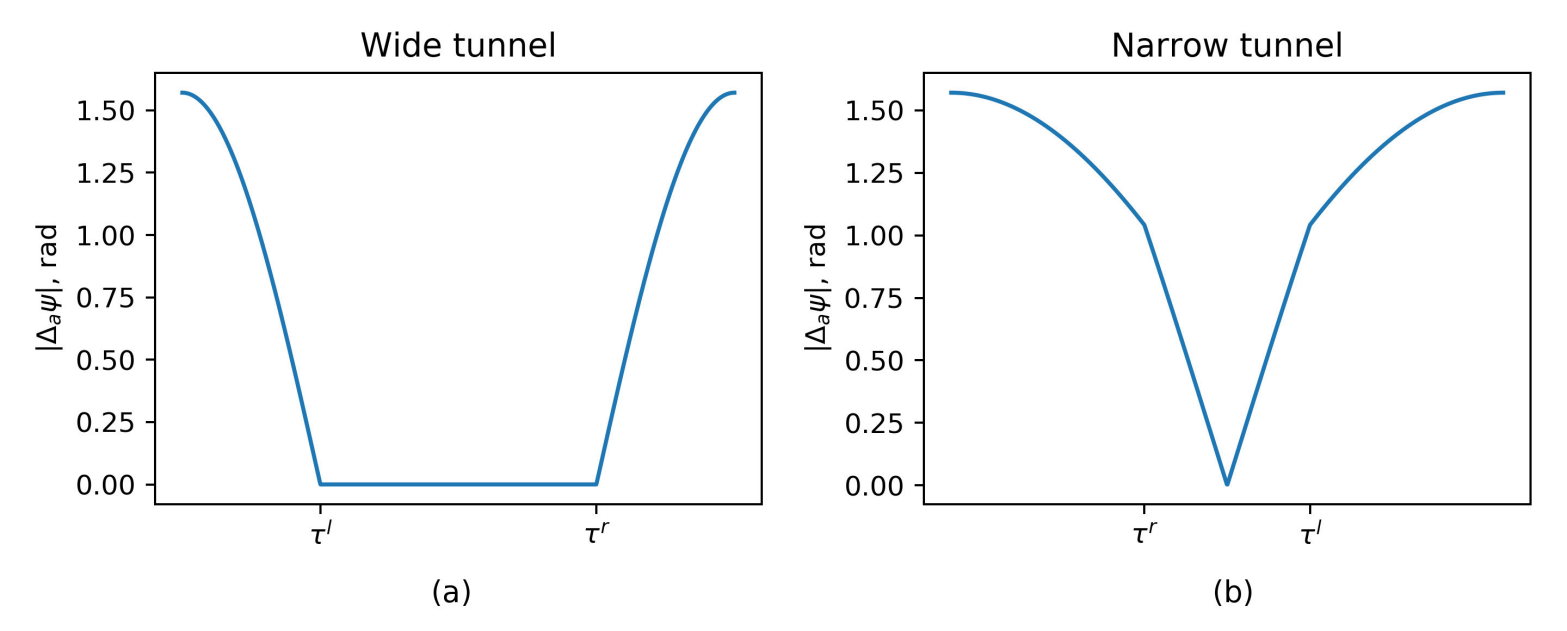
Yaw angle correction in wide and narrow tunnels. Notice that in the narrow tunnel case, the right wall reactive distance $\tau^{r}$ is at the left of the left wall reactive distance $\tau^{l}$. (**a**) Wide tunnel case. (**b**) Narrow tunnel case.

Using this formulation the potential field is zero for each sector outside of the reactive distance, beyond this point grows following a non-linear law until it reaches a π/2 angle correction near the wall. The resulting yaw correction is represented in Figure 7 for wide and narrow tunnels.

In general, as appointed by Özdemir in [24], directional approaches efficiently generate the directions outputs but do not take the dynamic of the vehicle into account. Also, angular correction is nonlinear by its very nature, the correction law is nonlinear and the vehicle dynamics can be nonlinear. An analytical calculation of stability it is not possible. Linearization is not considered as the control law has a large change in its gradient when the vehicle approaches a lateral wall. So simulation, real testing, and careful selection of the configuration parameters is needed.

The reactive distance should be selected to occupy a fraction of the tunnel, like in the wide tunnel configuration shown in Figure 7. This prevents destabilizing corrections in the center zone. In contrast, for the narrow tunnel configuration, obstacle avoidance algorithm will center the vehicle with respect to the tunnel with a proportional law in the central zone.

## 3. Hardware in the Loop Simulator

### 3.1. Architecture

The algorithm was validated using a using a hardware in the loop simulator (HIL). Having a simulator allows us to obtain, easily and at low cost, proper parameters for different tunnel topologies. Also, using a hardware or software in the loop simulator enables early detection of defects or bugs in the components.

**Figure 8:**
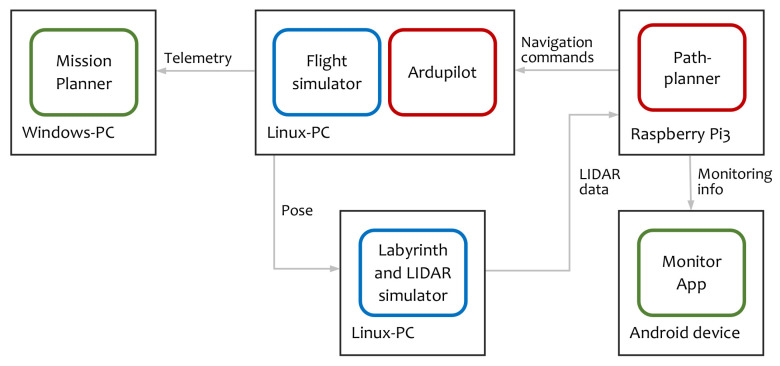
Hardware in the loop architecture. Red components are real unmanned aerial vehicle (UAV) software, blue components are simulators, and green components are monitoring applications.

The HIL architecture is represented in Figure 8.

The on-board software is composed of two parts: the flight controller and the path planner. The flight controller is a modified version of the Ardupilot [25], augmented with a new flight mode. In this new mode, the vertical behavior is to keep the vehicle stable at a constant height inside the tunnel. Current vehicle height is measured using two range finders, one pointing up, and the other pointing down. Using these measurements, a PID controller adjusts the vertical speed of the robot to maintain it at an intermediate height. The horizontal actions are to set the vehicle pitch angle to reach the desired horizontal speed and to adjust the vehicle yaw according to the navigation yaw error. The pitch angle is fixed for each speed, and the yaw is adjusted using a PID controller.

The path planner implements the algorithm described in this article. It is a soft real-time system and it is executed in the same hardware as in the real unmanned aerial vehicle (UAV). Therefore, the whole setup can be considered also as a hardware in the loop simulator.

The vehicle dynamics is computed using the Ardupilot software in the loop (SITL) simulator [26], named in Figure 8 as Flight simulator, and configured with a quadcopter model [27]. Internally it uses as engine JSBSim, an open-source, non-linear, six degrees of freedom aircraft simulator.

The labyrinth simulator receives in real-time the pose from the flight simulator and computes the LIDAR measurements. This labyrinth simulator was developed ad-hoc for this project and substitute the real sensor during the tests.

Labyrinth and LIDAR simulator are closely related as the LIDAR points are obtained performing a 2D line scan over the image of the labyrinth. In order to simulate realistic conditions, the LIDAR includes a noise model where any individual laser point can fail with a probability *p*, and the distance values are altered with white noise. The simulator uses the same scan range and angular point density as the reference sensor for this project, the Slamtech Mapper M1M1 [28].

### 3.2. Configuration

We have configured the HIL simulator with particular parameter values fitted to the features of the tunnels to be explored in our tests (see Section 4). These were designed to represent an unfavorable environment with narrow, about 1 m wide, and short mine passages. In this adverse context the vehicle needs to use low speeds, in the order of 0.1 m/s, a value close to other related works [9].

Hence, the base configuration is as follows. For the LIDAR simulation a setup, summarized in Table 1, with the same range and number of laser points as modern available sensors is used, the Slamtech Mapper M1M1 [28].

Error in measurements is included in the form of Gaussian noise and finally, individual laser points can fail with a 10% probability.

For the scan processing task, included in Table 2, the number of sectors used in the scan processing is 32. The maximum distance is set to be slightly smaller than the LIDAR range. Minimum points per sector prevents dust particles from generating a distance for a sector that is too short. The fraction of laser points used to average the distance of a sector is set to 1/3. Exploration vector distance is set relative to the typical tunnel width of the labyrinth. A distance that is too long avoids exploring short passages, and too short a distance allows exploring perpendicular to the tunnel. The typical tunnel width in the tests is around 1 m, so exploration distance is set to 2.5 m.

Related to the exploration vectors filtering, Table 3, the same angle limit is the relative angle between two exploration vectors to be considered the same vector. Too large a value in this parameter prevents exploring acute intersections, so a relatively low value is preferred. Hysteresis configuration is set to low values as the simulated scenario is very stable.

The navigation module setup is shown in Table 4. The continuation angle permits following a curved section in advance mode, at high speed, and without need to change to block or rotate mode. It also permits that the robot reattaches to an exploration vector when it is in block mode. An angle that is too large can cause a premature re-attachment and the robot can pass very close to an intersection corner. Block distance should be selected relative to the width of the tunnel, to permit the robot to approach a front wall and see other exploration vectors from there. Visited grid cell should be similar to the typical tunnel width and scan radius is set as explained in the visited zone tracking section.

To avoid trajectory oscillations, the reactive distance should be set to get a *wide tunnel* configuration represented in Figure 7. In the tests a too high reactive distance was used to provoke the *narrow tunnel* configuration and evaluate a more unstable situation. Near and far angles of avoidance sectors depend on the dynamic of the robot and were selected heuristically. Minimum valid points per sector avoid dust and transient problems. Obstacle avoidance configuration is summarized in Table 5.

The vehicle dynamics are configured in a copter model created by James Goppert. This model is the default one for the SITL simulator and it is available in the Ardupilot repository [27].

### 3.3. Algorithm Execution Time

A key feature for a real-time application of the path planner algorithm is its computing requirements. Besides the resilience to errors in the location estimates obtained by the SLAM, one of the advantages of the algorithm is its simplicity. It translates into low computation times. In the laser scan phase, a growth in the computing time when increasing the number of laser points is expected, while in the rest of the phases, a growth is expected when increasing the number of sectors.

**Figure 9:**
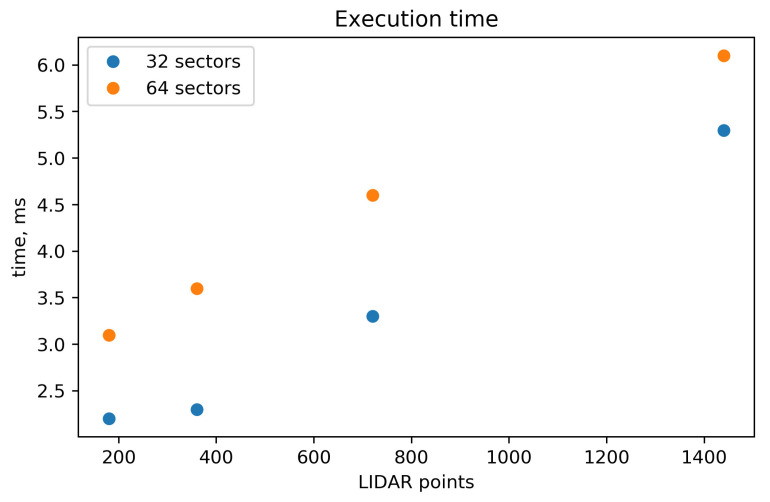
Algorithm execution time.

To evaluate these dependencies, algorithm execution times were measured for different numbers of LIDAR points and sectors. The algorithm was coded in Kotlin language and executed in a Raspberry Pi 3B+. The processor on this board is the BCM2835, with architecture ARMv7 (v7l) and central processing unit (CPU) frequency of 600 MHz. The execution times are shown in Figure 9.

The recorded data indicates a quasi-linear dependence between the execution time and the number of LIDAR points. However, as some execution time that does not depend on the number of LIDAR points exists, the times are bounded by a minimum value. As the number of LIDAR points grows, the relative difference between the times for 32 and 64 sectors becomes less significant. The reason is that in this case, the majority of the time is employed in the laser points processing, while when the number of the LIDAR points is small, the majority of the time is employed calculating the exploration vectors.

The configuration that can be considered the typical scenario comprises 32 sectors and 720 LIDAR points. The execution time for this characteristic case was 3.3 ms, which can be considered excellent and one of the big strengths of the algorithm.

## 4. HIL Simulations

### 4.1. Study Cases

A series of canonical situations is used to evaluate and explain the algorithm behavior in each case. Next, complete cyclic and acyclic labyrinths are considered. Table 6 and Table 7 summarize the cases.

**Figure 10:**
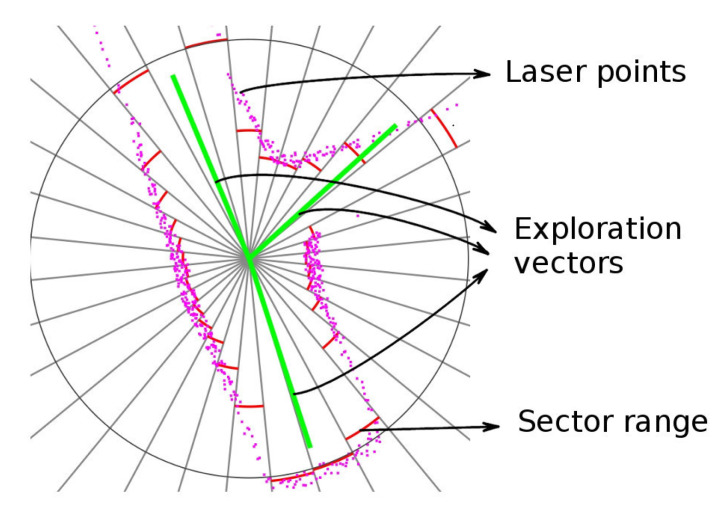
Interpretation of laser scan diagrams.

**Figure 11:**
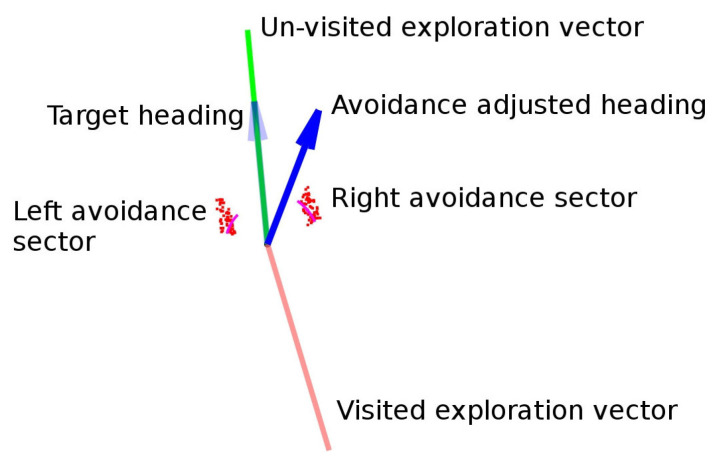
Interpretation of navigation diagrams.

**Figure 12:**
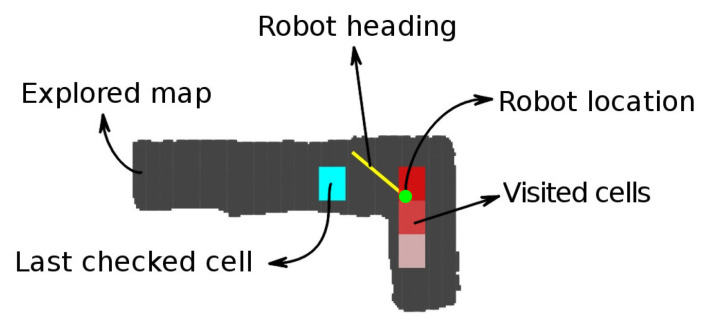
Interpretation of map diagrams.

Figure 10, Figure 11 and Figure 12 show the meaning of the symbols used in the rest of the section. In the laser scan diagrams all the exploration vectors are green, regardless of whether they were explored or not, while in the navigation diagrams, green is only used for unexplored vectors and red gradual color changes for explored vectors.

### 4.2. Canonical Situations Results

#### 4.2.1. Unexplored Corner

**Figure 13:**
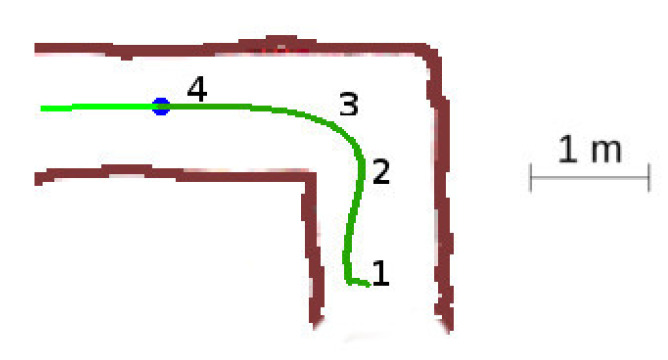
Unexplored corner situation.

In this situation, Figure 13, the robot starts the exploration of the labyrinth, so all the tunnels are unexplored, and it performs a 90-degree direction change.

**Figure 14:**
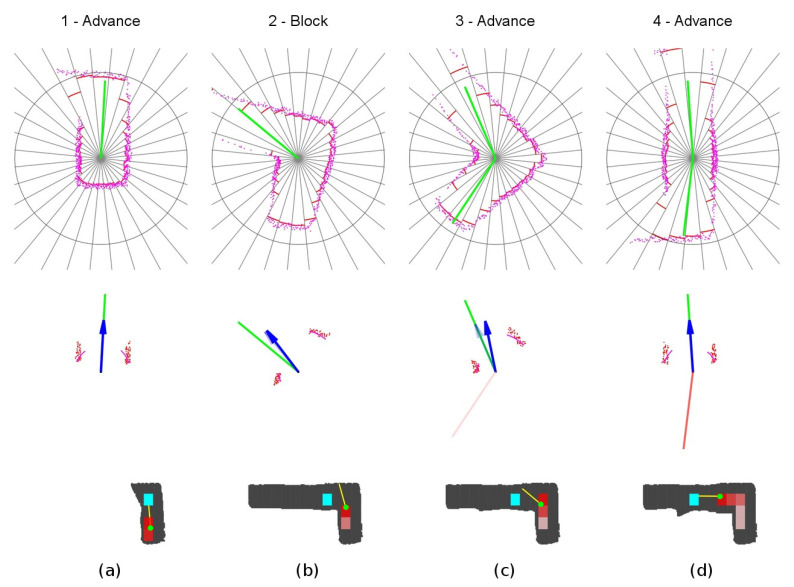
Unexplored corner secuence of events. (**a**) Location 1. (**b**) Location 2. (**c**) Location 3. (**d**) Location 4.

The detailed sequence is displayed in Figure 14. The robot starts at location 1, detects one suitable exploration vector, and sets the *advance mode* towards this direction. When reaching the front wall at location 2, it switches to the *block mode* and progress slowly towards the wall. At location 3, the robot detects a new exploration vector for which angle relative to the current yaw is not too large, so the robot reattaches to it. It changes to *advance mode* and continues into the final yaw. Notice the slight azimuth correction done by the obstacle avoidance component at step 3.

#### 4.2.2. Unexplored Three-Way Crossing

**Figure 15:**
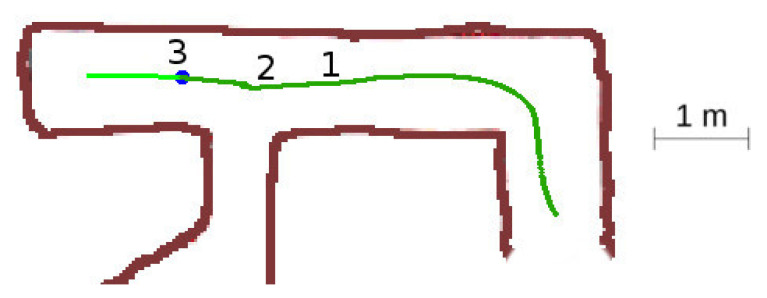
Unexplored three-way crossing situation.

In this situation, Figure 15, the robot detects a left-hand bifurcation, but it should continue forward as both, the intersection and the continuation vector, are unexplored.

**Figure 16:**
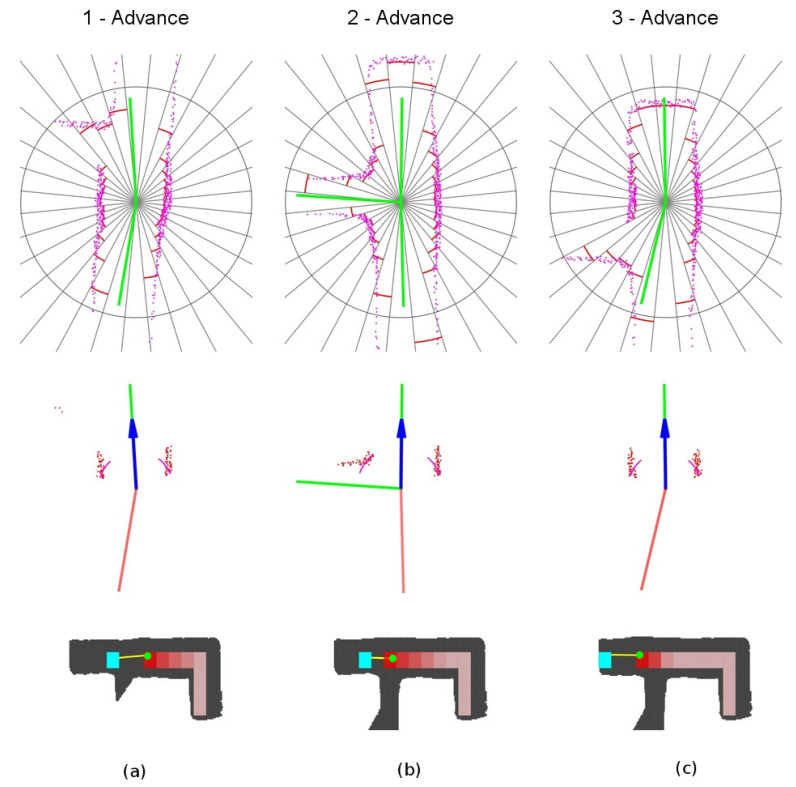
Unexplored three-way crossing secuence of events. (**a**) Location 1. (**b**) Location 2. (**c**) Location 3.

The detailed sequence is represented in Figure 16. The robot starts at point 1 with one unexplored vector in front of it and one explored vector behind. When it detects the third vector at point 2, it will choose the *continuation vector*, the most aligned with the current yaw. When it passes the intersection at point 3, the same situation as at point 1 remains, with one unexplored vector in front and one explored vector behind.

#### 4.2.3. Dead End

**Figure 17:**
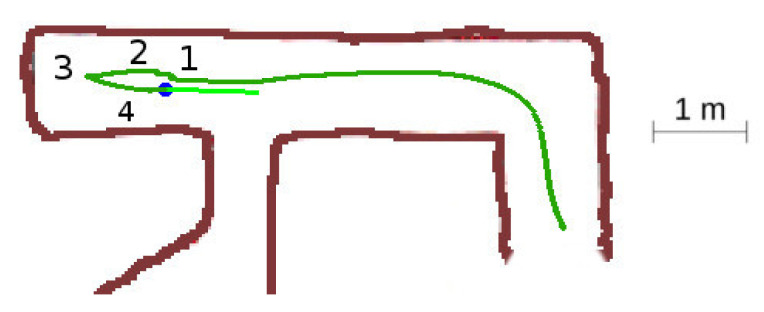
Dead end situation.

In this situation, Figure 17, the robot reaches a dead-end. It should approach the front wall at low speed to ensure that there are not other possible directions, and then turn back.

**Figure 18:**
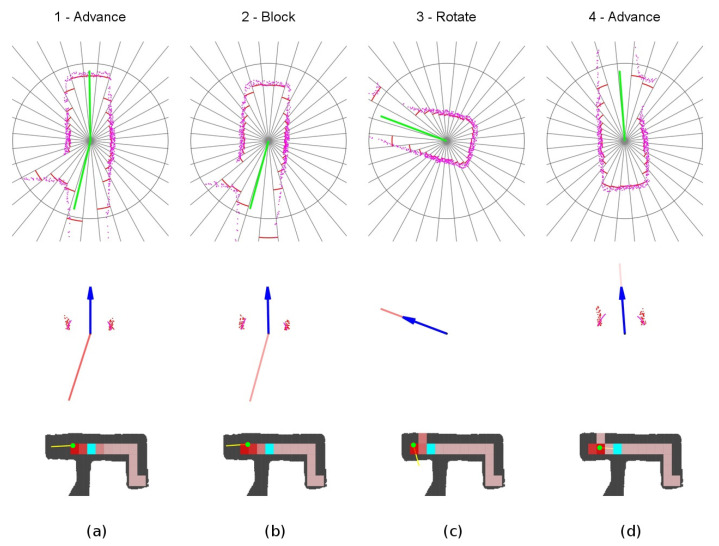
Dead end secuence of events (**a**) Location 1. (**b**) Location 2. (**c**) Location 3. (**d**) Location 4.

Detailed sequence is shown in Figure 18. The robot starts in advance mode with one explored vector behind it. Then, at location 2, it starts approaching the end wall at low speed in *block mode*. When close to the wall, at location 3, it will rotate to the only available exploration vector, that points backward of the starting direction.

#### 4.2.4. Explored Three-Way Crossing

**Figure 19:**
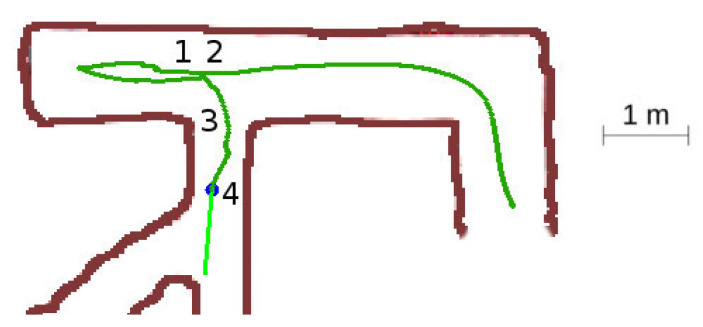
Explored three-way crossing situation.

This situation displayed in Figure 19 is similar at the beginning as the unexplored three-way crossing situation, but when it detects the bifurcation, there are two explored vectors and one unexplored, so the robot should take the bifurcation.

**Figure 20:**
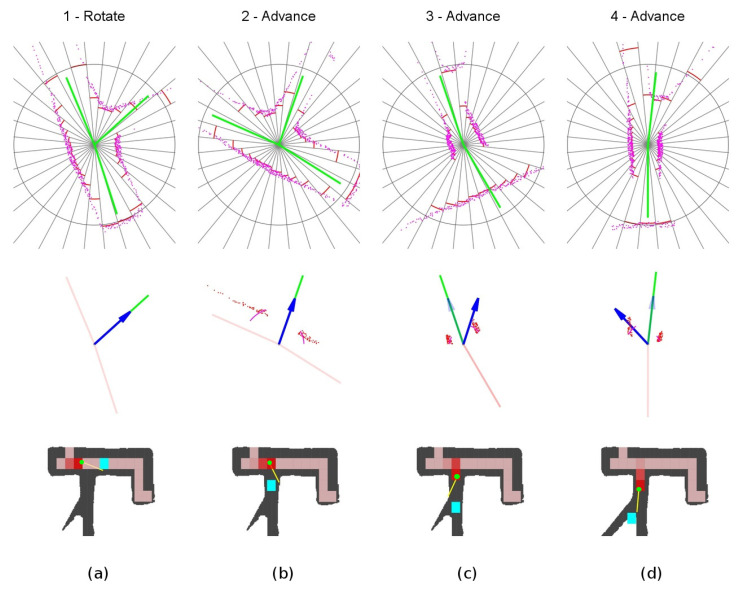
Explored three-way crossing secuence of events. (**a**) Location 1. (**b**) Location 2. (**c**) Location 3. (**d**) Location 4.

The detailed sequence is shown in Figure 20. The robot set the *rotate mode* and rotates the vehicle towards the third exploration vector. When already oriented, it switches to the *advance mode* at point 2. As the new corridor is very narrow, the obstacle avoidance is in the *narrow tunnel situation* represented in Figure 7. Notice the large yaw corrections, to the right at location 3 and to the left at location 4.

### 4.3. Gallery Labyrinth Cases

#### 4.3.1. Case 1: Acyclic Labyrinth

In the acyclic case, the robot explored the complete labyrinth and returned successfully to base. Some trajectory oscillations arose in the narrow vertical section due to the obstacle avoidance corrections.

**Figure 21:**
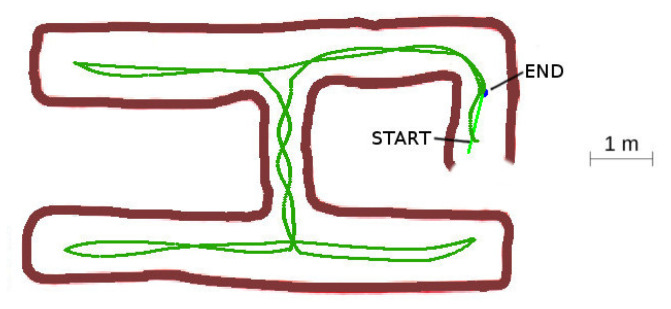
Acyclic labyrinth.

In the narrow central passage tunnel shown in Figure 21, some oscillating behavior arose due to the fixed value of the rective distance. It was set to a high value on purpose, to force the narrow tunnel configuration which is more unstable. Even in this adverse situation, the oscillations were slightly damped and disappeared when the vehicle reached the wider zone at the connected galleries. There is room for improvement of the algorithm using lateral motion combined with the yaw angle corrections to minimize these oscillations.

#### 4.3.2. Case 2: Cyclic Labyrinth

**Figure 22:**
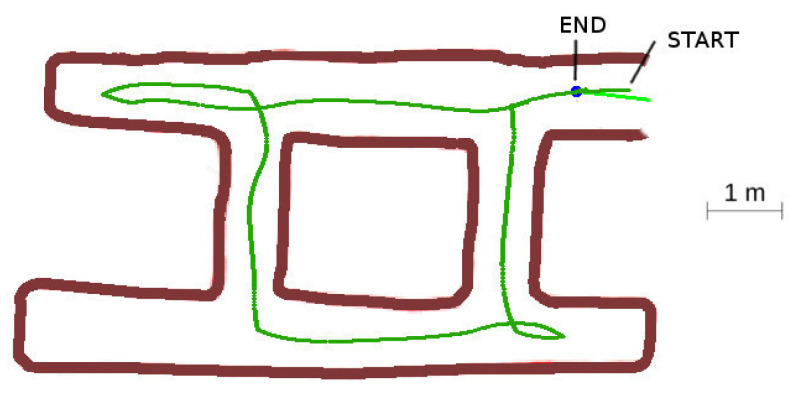
Cyclic labyrinth.

The cyclic labyrinth case, represented in Figure 22, shows the cost paid for the maze resolution without a global map. The bottom left corridor was not traveled. In this concrete case, the complete labyrinth was explored due to the LIDAR vision range from the bottom left intersection, but in other cases part of the labyrinth might be left unexplored. This is common in all of the algorithms that do not use a global logical map [2], but as the algorithm calculates the exploration vectors in the intersections, enough information can be saved to detect this circumstance. The robot returned correctly to base.

#### 4.3.3. Case 3: Cyclic Labyrinth with Curve Section

**Figure 23:**
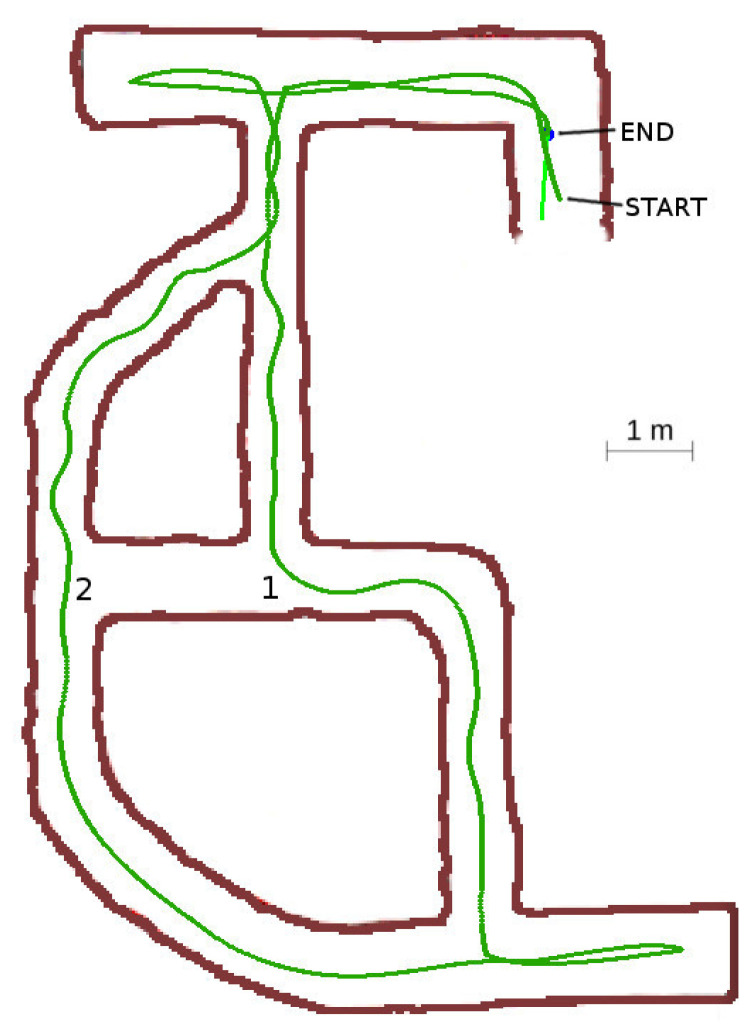
Cyclic labyrinth with curve section.

This labyrinth represents a more complex situation to evaluate the path planner algorithm response. The robot traveled the curved gallery at the bottom-left of Figure 23 in *advance mode* the entire time, without the need to change to the block or rotate modes as the curve could always be followed using a *continuation vector*. The resulting path used to explore and return to base was optimum, although some oscillations arose in the narrowest sections.

A very interesting situation happened at location 1. When the robot was at this point, the exploration radius δ was long enough to test the visited grid at location 2. The robot detected that all the gallery was already explored and decided to turn right at the intersection.

## 5. Discussion

Next, we will evaluate the performance of the developed path planner algorithm. It is performed by computing the distance and the time required for the vehicle in the exploration. Both are compared with respect to a reference case, which reflects the most favorable path plan to explore a particular labyrinth. In particular, it is defined as that requiring the minimum total distance and time of the vehicle as next defined, although its ideal nature makes it not physically realizable.

The traveled distance reflects two sources of inefficiency. The first is derived from making bad routing decisions, the second one is caused by traveling in curved trajectories instead of straight lines along the tunnels.

With respect to the routing decisions, in all of the cases, the navigation logic performed as expected and the used routing was optimal. In case 2 with the cyclic labyrinth one tunnel was left unexplored, so we will consider the reference case also without this section.

To calculate the ideal distance, each reference case is composed of straight lines along the tunnels, turns with a radius of 0.7 m at the intersections and a 1 m margin at the dead ends of the tunnels.

Table 8 shows the path efficiency of the algorithm with respect to the reference case. The excess in the traveled distance is small in all the cases and is caused by the curved paths and because the dead-end distances sometimes are smaller than 1 m. Taking this into account, we are really evaluating the algorithm and vehicle dynamics combination.

To evaluate the performance of the travel time, we assume for the reference case, that all of the path is traveled at high speed except near the walls, and that there is no need for any rotation time. When approximating a front wall, one meter at low speed is applied. This is the best case that we can consider because the condition of not needing turns is possible in other algorithms as a multi-copter can move in every direction without the need to turn into it.

The results of time performance are included in Table 9. As expected, the relative excess in time is greater than in distance because we spent time in rotations.

**Figure 24:**
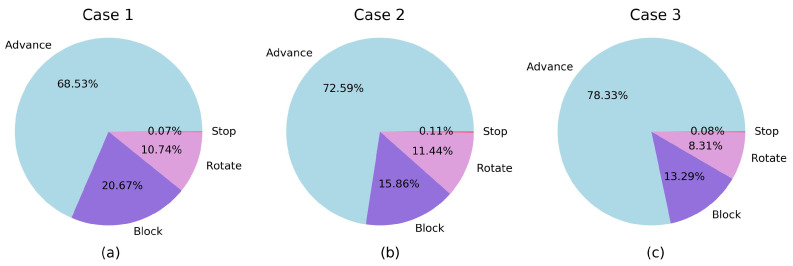
Relative time elapsed in each mode. (**a**) Case 1. (**b**) Case 2. (**c**) Case 3.

Time was divided between the different modes as shown in Table 10 and Figure 24. The majority of the time was used in the advance mode. The block mode impact in the total used time can be minimized using a smaller block distance or using a slow speed that is not so low. The rotation time is around 10% so it can be considered appropriate and with low impact in the total time.

The time elapsed in each mode reflects aspects related to the topology of the labyrinths. In case 3, the percentage in advance mode is higher than in the other cases, as expected for a bigger labyrinth with longer galleries. As a consequence, the excess in time with respect to the reference case is lower for this case. Although case 1 and case 2 are labyrinths of similar size, there is more time in block mode in case 1; this reflects that the labyrinth of case 1 has one more dead-end than the case 2 one (the difference is the dead-end that is left unexplored in case 2). Accordingly, this results in a higher excess in time in case 1 with respect to the reference case. The times spent in stop mode are minimum for all the cases. Finally, the time spent in the rotate mode depends on the total angular rotation needed to explore the different labyrinths, with a lower value in case 3 caused by the aforementioned effect of being a bigger labyrinth with longer galleries.

## 6. Conclusions

Within this work we constructed a path planning solution to explore underground environments, specifically a large set of single-level mining galleries, using aerial robots (typically multicopters). Due to the lack of navigation signals and radio communications, the exploration is performed by equipping the vehicle with a LIDAR sensor with integrated SLAM capabilities. A path planner has been then developed considering a dynamic planning approach based on exploration vectors. Also, a required global awareness has been developed with the aid of a visited zone grid combined with an exploration radius. It prevents the UAV from being trapped in a loop. One of the main advantages of this solution is that it avoids maintaining a logical map, difficult to build from noisy and unstable LIDAR scans, and that may be broken if the error in position estimation becomes large. Furthermore, the grid and the rest of the algorithm can be implemented easily using only static memory, which is the preferred option for real-time applications. Another advantage of the proposed method is that, although the grid can be corrupted if there is a significant error in the location estimates, it can never be disrupted, and the navigation module will continue making decisions. In this limiting case there is no guarantee that the robot returns to base, but the trend is that it does, as the navigation module always looks for the oldest explored direction. Another difficulty, common to local approaches, is that it cannot be ensured that all the tunnels of cyclic mine galleries are explored. However, there is enough information recorded to detect non explored tunnels that can be analyzed a posteriori.

Finally, to facilitate the navigation along narrow mine passages, avoiding vehicle damage, an additional obstacle avoidance capability has also been incorporated into the robot flight controller. It just relays the output of the laser scan processing and can be tuned by selecting the proper parameters, like the avoidance sector and the reactive distance, according to the expected tunnel characteristics. The designed path planner algorithm has been validated by means of a hardware-in-the-loop (HIL) simulator which was designed for the purpose for this research. The tests consisted of exploring some canonical cases and labyrinths. The derived results prove that the decision logic worked as expected. Its performance efficiency has also been evaluated with HIL simulations in cyclic and acyclic labyrinths. We characterized it in terms of traveled distance and time used for the exploration with respect to an ideal reference case. It turned out that the excess in the traveled distance and total time was small. Then, the result is a very fast, real-time, and static memory capable algorithm.

According to the results presented in this paper, the proposed combination of aerial robot and path planner algorithm can be considered as a suitable solution for the autonomous exploration of underground mine galleries. Further developments are expected to include additional capabilities, e.g., some kind of 3D navigation to change from a mine level to another one through vertical passages. They will require an extension of the path planner algorithm, as well as an improved trajectory control of the aerial vehicle, exploiting its 6 DoF dynamics. It would broaden the utility of our solution to more applications, from mapping the tunnels to carrying specialized payloads (e.g., to detect poisoning gases), optimizing operational costs, and avoiding exposing human lives to any danger.

## Figures and Tables

**Table 1:** Simulated LIDAR parameters.

Maximum range	12 m
Number of laser points	720
Range noise, typical deviation	0.5 m
Laser point fail probability	0.1

**Table 2:** Scan processing configuration.

Number of sectors, N	32
Maximum distance, $\rho_{max}$	10 m
Minimum points per sector, $J_{min}$	5
Fraction of the seed sector to check continuation	0.6
Fraction of laser points to average the distance	0.33
Minimum exploration vector distance	2.5 m

**Table 3:** Exploration vectors filter.

Same angle limit	20${}^{\circ }$
Hysteresis, active limit	4
Hysteresis, de-active limit	2
Hysteresis, anti-windup limit	5
Distance low-pass filter alpha, $\alpha_{1}$	0.4
Azimuth low-pass filter alpha, $\alpha_{2}$	0.4

**Table 4:** Navigation module configuration.

Continuation angle	40${}^{\circ }$
Block distance	1 m
Visited grid cell size, *s*	0.8 m
Scan radius, δ	2.0 m

**Table 5:** Obstacle avoidance configuration.

Reactive distance, τ	0.7 m
Near angle at low speed, $\Gamma_{1}^{L}$	10${}^{\circ }$
Far angle at low speed, $\Gamma_{2}^{L}$	35${}^{\circ }$
Near angle at high speed, $\Gamma_{1}^{H}$	20${}^{\circ }$
Far angle at high speed, $\Gamma_{2}^{H}$	50${}^{\circ }$
Minimum valid points per sector	5

**Table 6:** List of canonical situations.

Unexplored corner
Unexplored three-way crossing
Dead end
Explored three-way crossing

**Table 7:** List of complete labyrinth cases.

Case 1	Acyclic labyrinth
Case 2	Cyclic labyrinth
Case 3	Cyclic labyrinth with curve section

**Table 8:** Traveled distance vs. ideal case (Units: meters).

	Ideal Distance	Traveled Distance	Excess
Case 1	42.83	49.17	6.34 (14.8%)
Case 2	28.59	32.02	3.43 (12.0%)
Case 3	59.64	67.99	8.35 (14.0%)

**Table 9:** Used time vs. ideal case (Units: seconds).

	Ideal Time	Used Time	Excess
Case 1	606.7	745.3	138.6 (22.8%)
Case 2	396.3	459.7	63.4 (16.0%)
Case 3	770.4	891.9	121.5 (15.8%)

**Table 10:** Time elapsed in each mode (Units: seconds).

	Advance	Block	Rotate	Stop
Case 1	509.9	153.8	79.9	0.5
Case 2	333.7	72.9	52.6	0.5
Case 3	698.6	118.5	74.1	0.7

## Abbreviations

The following abbreviations are used in this manuscript:

APF	Artificial potential field
CPU	Central processing unit
DARPA	Defense Advanced Research Projects Agency
DoF	Degrees of freedom
GNSS	Global navigation satellite system
HIL	Hardware in the loop
INS	Inertial system unit
LIDAR	Laser imaging detection and ranging
MDPI	Multidisciplinary Digital Publishing Institute
SITL	Software in the loop
SLAM	Simultaneous localization and mapping
SONAR	Sound navigation and ranging
UAV	Unmanned aerial vehicle
